# Investigating Drought Tolerance in Chickpea Using Genome-Wide Association Mapping and Genomic Selection Based on Whole-Genome Resequencing Data

**DOI:** 10.3389/fpls.2018.00190

**Published:** 2018-02-19

**Authors:** Yongle Li, Pradeep Ruperao, Jacqueline Batley, David Edwards, Tanveer Khan, Timothy D. Colmer, Jiayin Pang, Kadambot H. M. Siddique, Tim Sutton

**Affiliations:** ^1^School of Agriculture, Food and Wine, The University of Adelaide, Adelaide, SA, Australia; ^2^School of Agriculture and Food Sciences, The University of Queensland, Brisbane, QLD, Australia; ^3^School of Biological Sciences, The University of Western Australia, Perth, WA, Australia; ^4^The UWA Institute of Agriculture, The University of Western Australia, Perth, WA, Australia; ^5^UWA School of Agriculture and Environment, The University of Western Australia, Perth, WA, Australia; ^6^South Australian Research and Development Institute, Adelaide, SA, Australia

**Keywords:** drought tolerance, genome-wide association mapping, genomic selection, chickpea, whole-genome resequencing, auxin

## Abstract

Drought tolerance is a complex trait that involves numerous genes. Identifying key causal genes or linked molecular markers can facilitate the fast development of drought tolerant varieties. Using a whole-genome resequencing approach, we sequenced 132 chickpea varieties and advanced breeding lines and found more than 144,000 single nucleotide polymorphisms (SNPs). We measured 13 yield and yield-related traits in three drought-prone environments of Western Australia. The genotypic effects were significant for all traits, and many traits showed highly significant correlations, ranging from 0.83 between grain yield and biomass to -0.67 between seed weight and seed emergence rate. To identify candidate genes, the SNP and trait data were incorporated into the SUPER genome-wide association study (GWAS) model, a modified version of the linear mixed model. We found that several SNPs from auxin-related genes, including auxin efflux carrier protein (PIN3), p-glycoprotein, and nodulin MtN21/EamA-like transporter, were significantly associated with yield and yield-related traits under drought-prone environments. We identified four genetic regions containing SNPs significantly associated with several different traits, which was an indication of pleiotropic effects. We also investigated the possibility of incorporating the GWAS results into a genomic selection (GS) model, which is another approach to deal with complex traits. Compared to using all SNPs, application of the GS model using subsets of SNPs significantly associated with the traits under investigation increased the prediction accuracies of three yield and yield-related traits by more than twofold. This has important implication for implementing GS in plant breeding programs.

## Introduction

Chickpea (*Cicer arietinum* L.) is ranked second after soybean in terms of global legume production, reaching ∼13 million tons in 2014 (FAOSTAT 2017). Consisting of 25% of the total exports worldwide, Australia was the second-largest producer and the largest exporter of chickpea in 2014 (FAOSTAT 2017). Chickpea is an important component of the farming system in Australia, serving as a disease break crop and nitrogen fixer (Knights et al., 2009; Siddique et al., 2013). Chickpea seed is a rich source of protein, essential minerals, and dietary fiber (Bar-El Dadon et al., 2017).

Drought is one of the most important constraints limiting yield potential in cereal and legume crops. Significant differences in terms of drought tolerance, measured by yield reduction, were observed among legume species in a meta-analysis of over 100 studies with chickpea ranking seventh among 13 legume species (Daryanto et al., 2015). There are generally two types of drought: transient drought and terminal drought. Transient drought is a short-term water deficit that can be relieved by precipitation and can occur at any stages of the growing season. Terminal drought is an unrelieved water deficit that terminates the reproductive growth of the plant. Terminal drought is very common in semi-arid tropics (South Asia, north-east Australia) and Mediterranean-type climates such as southern Australia. More than 80% of the world chickpea production is located in South Asia and north-east Australia. Australia has experienced severe drought events from the late 1990s to mid 2000s known as “the Millennium drought.” As a consequence, the total production of irrigated rice and cotton fell by 99 and 84% during 2002 and 2009, respectively (van Dijk et al., 2013).

With current climate change projections, extremely hot weather will become more frequent and rainfall will be more erratic in Australia and other regions of the world (Hennesy et al., 2010; Foyer et al., 2016). The reproductive stage of growth is usually the most critical phase influencing grain yield in crops. It is well documented that drought stress during pod filling can lead to pod abortion thus reducing the number of seeds per plant (Leport et al., 1999; Fang et al., 2010; Pang et al., 2017). In a glasshouse experiment, seed yield declined by 85% in chickpea plants exposed to terminal drought at the early podding stage, relative to well-watered plants (Pang et al., 2017). There is an urgent need to develop chickpea varieties that are drought resilient.

Chickpea has a relatively small genome size of 730 Mb, compared to other food legumes such as lentil (*Lens culinaris* L.) and faba bean (*Vicia faba* L.). Thanks to the advance of next-generation sequencing (NGS) technology and a relatively small genome size, chickpea has well-developed genomic resources. Chickpea reference genomes for kabuli, desi, and wild *Cicer* species are available and there are ongoing efforts to improve the assemblies and annotation of the genomes (Jain et al., 2013; Varshney et al., 2013; Ruperao et al., 2014; Parween et al., 2015; Gupta et al., 2017). Whole-genome resequencing (WGRS) has emerged as one of the best methods for genome-wide association studies (GWAS) due to its potential to discover a large amount of sequence variants [single nucleotide polymorphisms (SNPs), Indel, CNV] in a cost effective manner. A recent study using this method narrowed down a major QTL for ascochyta blight resistance in chickpea (Li et al., 2017). Using WGRS data, several genomic regions were identified under positive selection for plasticity for yield, nitrogen fixation, and δ13C in chickpea under drought and/or heat conditions in the field (Sadras et al., 2016).

To identify QTL/genes associated with drought tolerance in chickpea, different forward-genetic approaches using various molecular markers have been used. A recent study using bi-parental mapping population (ILC588 × ILC 3279) and sparse simple sequence repeat (SSR) markers, identified 15 and 93 QTL associated with different drought-related traits (Rehman et al., 2011; Hamwieh et al., 2013). However, the resulting large QTL intervals have limited practical application in breeding. A “QTL-hotspot” region on chromosome 4 was identified using traditional QTL analysis with SSR and GBS (genotyping-by-sequencing) markers (Varshney et al., 2014b; Jaganathan et al., 2015). This region was further fine mapped to a ∼300 kb region, which contains 26 genes using a QTL bin-mapping approach and gene enrichment analysis by adding more SNPs (Kale et al., 2015). The authors also tested 12 genes for differential gene-expression profiling using real-time PCR. Under drought condition, several genes had higher gene-expression levels in the resistant line than in susceptible lines, including E3 ubiquitin ligase, serine/threonine protein kinases, and homocysteine S-methyltransferase. Another study, employing the GWAS approach, discovered over 200 markers associated with drought-related traits using SSR, DArT, and SNP markers (Thudi et al., 2014). The results, albeit needing further validation, are promising for marker-assisted selection. However, the development of these molecular markers is labor-intensive and not cost-effective.

One of the challenges of marker-assisted selection is how to pyramid numerous markers with small effect size, particularly for complex traits such as yield under drought environments (Collins et al., 2008). As such, genomic selection (GS), also known as genome-wide selection, was proposed as an alternative method for marker-assisted selection for complex traits (Meuwissen et al., 2001). GS uses information from all of the markers to estimate the breeding value of plants, thus eliminating the complicated pyramiding process in marker-assisted selection. This approach is more relevant to breeding programs as it can help select the best parents for crossing and reduce the cost and time of a standard breeding cycle; thus it has been adopted rapidly by many livestock and crop breeding programs (Hayes et al., 2009a; Meuwissen and Goddard, 2010; Crossa et al., 2014). Traditionally, animal scientists estimated breeding values by best linear unbiased prediction (BLUP) using the additive-genetic relationship matrix obtained from pedigree information (Henderson, 1975). Thanks to advances in genotyping and NGS technology, a large amount of molecular markers can be obtained at a relatively low cost (Davey et al., 2011; Elshire et al., 2011; Poland and Rife, 2012). The genomic estimated breeding values can be estimated more accurately using the ridge regression BLUP (RR-BLUP) model which replaces the pedigree matrix (**A** matrix) with the genomic relationship matrix (**G** matrix), which is obtained from genome-wide markers (VanRaden, 2008; Hayes et al., 2009b). A simulation study in barley showed that GS was better than phenotypic selection when the traits had low heritability and the training population was large enough. Ziyomo and Bernardo (2013) demonstrated in a real experiment that GS is superior to indirect phenotypic selection using secondary traits for improving drought tolerance of maize. However, a recent GS study in chickpea showed that prediction accuracies of yield under rainfed environments were much lower than under irrigated environments (Roorkiwal et al., 2016). A similar observation was reported in synthetic wheat, posing a challenge to improving drought tolerance using GS (Jafarzadeh et al., 2016). A new method is needed to increase prediction accuracy, particularly when applied to drought-stressed environments. The objectives of this study were to: (1) identify candidate genes/SNPs significantly associated with yield and yield-related traits under drought stressed environments using GWAS approaches; and (2) investigate whether incorporation of the GWAS result can increase prediction accuracy.

## Materials and Methods

### Plant Materials and Field Experiments

Plant materials included 13 Australian released varieties and 119 Australian and Indian-derived breeding lines, which were selected for yield potential and adaptation to drought-prone environments. The field experiments are described in detail by Pang et al. (2017). Briefly, chickpea accessions were planted in plots (6 × 1.5 m) in Western Australia at one site in 2012 and two sites in 2013. There were three replicates for each site. Rainfall at the three sites during the growing seasons ranged from 196 to 230 mm, and no irrigation was supplied. Twelve traits were measured: grain yield per ha (GY), hundred seed weight (100SW), seed number per plant (SN), empty pod ratio (EPR), harvest index (HI), biomass dry weight (DW), flowering time score (FT), podding time score (PT), maturity score (MA), emergence score (EM), early vigor score (EV), and plant height (PH). Five plants per plot were randomly cut at ground level to measure SN, EPR, HI, and DW. Scores for FT, PT, MA, EM, and EV were on a 1–9 scale.

### Phenotypic Analysis

The three sites were first analyzed separately for each trait by fitting a linear mixed model (LMM), which included spatial effects (row and column effects). The resulting best linear unbiased estimator (BLUE) values for each genotype were used to fit a multiple-environment LMM in which environments were treated as random effects. Statistical significance of fixed and random effects were assessed using Wald’s test (Wald, 1943) and the likelihood ratio test, respectively (Van Belle et al., 2004). The resulting BLUE values were subsequently used for GWAS analysis. Broad-sense heritability (*h*^2^) was estimated using the following formula:

$$\begin{array}{c} {\hat{h}}^{2}\;={\hat{\sigma }}_{g}^{\;\;2}/({\hat{\sigma }}_{g}^{\;\;2}+{\hat{\sigma }}_{\mathrm{ge}}^{\;\;\;\;2}/t+{\hat{\sigma }}_{e}^{\;\;2}/rt) \end{array}$$

where ${\hat{\sigma }}_{g}^{\;\;2}$, ${\hat{\sigma }}_{\mathrm{ge}}^{\;\;2}$, and ${\hat{\sigma }}_{e}^{\;\;\;\;2}$ denote genotypic variance, genotype × environment interaction variance, and experimental error variance, respectively. *t* and *r* are the numbers of environments and replications within an environment, respectively. All phenotypic analysis was done using GenStat, 16th edition.

### WGRS and SNP Discovery

DNA of the 132 genotypes was extracted from young leaf tissues using the Qiagen DNeasy Plant Mini Kit following the manufacturer’s instruction. Paired-end sequencing libraries were constructed using the TruSeq library kit for each genotype with an insert size of 500 bp. The procedure was implemented according to the Illumina manufacturer’s instruction. Paired-end short reads (150 bp) were generated using the Illumina HiSeq 2000 platform. Sequence data is available from the NCBI Short Read Archive under BioProject accession PRJNA375953. Paired-end reads for each genotype were trimmed, filtered, and mapped to the kabuli reference genome 2.6.3^1^ using SOAP2. Homozygous SNPs were called using the SGSautoSNP pipeline (Lorenc et al., 2012).

### Population Structure and Linkage Disequilibrium

To correct for confounding effects in the association studies, population structure was estimated based on 144,777 SNPs (MAF > 0.05) using ADMIXTURE (v1.23) software (Alexander and Lange, 2011). Similar to the popular software STRUCTURE, ADMIXTURE uses a model-based algorithm to estimate the ancestry of unrelated individuals. The number of underlying population groups (K) was estimated from 1 to 10 using the maximum likelihood estimation approach with a fast numerical optimization algorithm. Cross-validation method of Alexander and Lange (2011) was used to determine the most likely number of population group (K). Linkage disequilibrium (LD) was measured by the parameter *r*^2^ using SNPs with high confidence (minimum five reads per genotype). An *r*^2^ = 0.2 was used as a threshold to determine LD extent. The method to estimate the LD-decay curve under the mutation-drift-equilibrium model was described in detail in Li et al. (2011).

### Genome-Wide Association Mapping

Genome-wide association analysis was done using BLUE values of the 132 genotypes with 12 traits and 144,777 SNPs (MAF > 0.05). Adjusting the confounding effects of population structure and kinship, the SUPER GWAS method, implemented in the GAPIT software, was used to estimate each SNP effect (Lipka et al., 2012; Tang et al., 2016). This method can increase statistical power by estimating kinship matrix with a subset of markers which are not in LD with the testing marker (Wang et al., 2014). The kinship matrix was estimated using the VanRaden method and later compressed to its optimum groups using the P3D method to speed up computation time. Default parameters of the SUPER model were used: sangwich.top = “MLM,” sangwich.bottom = “SUPER,” LD = 0.1. The significant *p*-value cut-off was set as *p* = 3.45e-07, equivalent to the α level of 0.05 after Bonferroni correction. The two genes flanking the significant SNP are reported.

### Genomic Selection

Genomic predictions were performed using three different models: RR-BLUP, Bayesian least absolute shrinkage and selection operator (Bayesian LASSO or BL), and Bayesian ridge regression (BRR). The RR-BLUP model is written as:

$$\begin{array}{c} {y=\mu 1}_{n}+Zg+e,\;{e\sim N(0,I\sigma }^{2}),, \end{array}$$

where *y* is the adjusted entry means of phenotypes, μ is the overall mean, 1*n* denotes *n* × 1 vector of 1s, **Z** is an incident matrix for random genotype effect, and *g* is genotype effect with normal distribution N (0, ${G\sigma }_{g}^{2}$), where **G** is the genomic relationship matrix obtained from markers (VanRaden, 2008). The markers included all 147,777 markers or a subset of markers selected based on different levels of *p*-value from GWAS.

The general structure of the two Bayesian linear regressions BL and BRR can be written as,

$$\begin{array}{c} p(\mu ,\beta {,\sigma }^{2}|y,\theta ), \end{array}$$

the posterior probability of unknown parameters includes overall mean μ, marker effect β, and its variance σ^2^, given the data y and hyperparameters **θ**. Estimates of these unknown parameters are obtained by solving the optimization problem and adding a penalty function to **β**. For BRR, the same Gaussian prior was assigned to **β**, resulting in the same shrinkage for all markers. For BL, a Bayesian version of the least absolute shrinkage and selection operation (Tibshirani, 1996) was introduced in the penalty function of **β**, resulting in greater shrinkage of markers with small effects and less shrinkage of markers with large effects. BL has a special feature of both variables selection and shrinkage, whereas BRR only shrinks variables. The detailed similarities and differences between genomic prediction models are reviewed by de Los Campos et al. (2013). The R package synbreed was used to fit the three models (Wimmer et al., 2012).

A fivefold cross-validation was performed to evaluate the prediction performance of the three models. The whole dataset was randomly divided into five mutually exclusive subsets, four of which formed the training set for fitting the model and the fifth was used as a test set. This process was repeated ten times, resulting in 50 cross-validations. Predictive abilities were calculated as Pearson’s correlation coefficient between the predicted values and observed phenotypic values of the test set. An average predictive ability of 50 cross-validations was reported.

## Results

### Yield and Yield-Related Traits

In total, 12 traits including phenology, yield, and yield components were measured. Multiple-environment linear mixed-models were fitted to obtain BLUE values for each genotype (**Table 1** and Supplementary Figure S1). The genotypic effect of SN was significant at an alpha level of 0.05 while the other 11 traits were highly significant at an alpha level of 0.001 (**Table 1**). Heritabilities (*h*^2^) of the 12 traits ranged from 0.11 for GY to 0.91 for 100SW. Many traits showed highly significant correlations, ranging from 0.83 between GY and DW to -0.67 between 100SW and EM (**Table 2**). GY was positively correlated with 100SW, SN, DW, FT, PT, EM, EV and negatively correlated with EPR, MA, and PH. 100SW was positively correlated with FT, DW, MA and negatively correlated with SN, PT, and EM. GY has a highly positive correlation with DW (*r* = 0.83), which is not surprising, given that a strong and healthy plant with sufficient biomass is advantageous under drought to retain yield (Hamwieh et al., 2013; Kashiwagi et al., 2013). PT and MA were negatively correlated (-0.64), which appears counterintuitive. Due to the low temperatures in early spring in Australian environments, some genotypes originating from India with an early podding trait, aborted their early onset pods which is reflected in the negative correlation between PT and EPR (i.e., the earlier the podding time, the higher the EPR). This supports the observation that chickpea plants need to set pods within a fairly narrow window to optimize yield in Australian environment.

**Table 1:** BLUE values (minimum–maximum), genotypic effect, and heritabilities (*h*^2^) of 12 traits obtained from a multi-environment LMM.

Traits	No. of genotypes	Mean	Minimum	Maximum	Wald’s test for genotypic effect	*H*^2^
GY (kg)	132	1027.11	623.19	1264.75	*p* < 0.001	0.11
100SW (g)	132	20.00	15.00	37.61	*p* < 0.001	0.91
SN	93	21.96	11.91	32.28	*p* = 0.015	0.32
EPR	59	0.31	0.14	0.46	*p* < 0.001	0.52
HI	93	0.38	0.28	0.46	*p* < 0.001	0.51
DW (g)	93	9.81	6.14	18.02	*p* < 0.001	0.49
FT	132	5.36	1.98	9.56	*p* < 0.001	0.70
PT	132	5.31	0.55	9.27	*p* < 0.001	0.72
MA	132	5.97	4.61	10.04	*p* < 0.001	0.25
EM	132	7.95	5.68	8.88	*p* < 0.001	0.49
EV	132	6.13	3.65	7.93	*p* < 0.001	0.65
PH (cm)	62	53.62	42.88	61.84	*p* < 0.001	0.64

*GY, grain yield per ha; 100SW, hundred seed weight; SN, seed number per plant; EPR, empty pod ratio; HI, harvest index; DW, biomass dry weight; FT, flowering time score; PT, podding time score; MA, maturity score; EM, seed-emergence score; EV, early vigor score; PH, plant height.*

**Table 2:** Correlation matrix of the 12 traits.

	GY	100SW	SN	EPR	HI	DW	FT	PT	MA	EM	EV	PH
GY	–											
100SW	0.45***	–										
SN	0.47***	–0.36**	–									
EPR	–0.37**	–0.12	–0.22	–								
HI	0.13	–0.27*	0.56***	0.02	–							
DW	0.83***	0.67***	0.15	–0.33*	–0.35**	–						
FT	–0.43***	0.42***	–0.14	–0.55***	–0.28*	0.43***	–					
PT	0.58***	–0.42***	0.27*	0.58***	0.41**	–0.40**	–0.65***	–				
MA	–0.53***	0.70***	–0.17	–0.35**	–0.47***	0.73***	0.54***	–0.64***	–			
EM	0.42***	–0.67***	0.20	0.25	0.27*	–0.59***	–0.29*	0.38**	–0.59***	–		
EV	0.29*	–0.20	–0.03	0.65***	0.03	–0.29*	–0.60***	0.55***	–0.37**	0.25	–	
PH	–0.22	0.25	–0.56***	0.18	–0.58***	0.08	0.11	–0.10	0.28*	–0.27*	0.25	–

*GY, grain yield; 100SW, hundred seed weight; SN, seed number; EPR, empty pod ratio; HI, harvest index; DW, biomass dry weight; FT, flowering time score; PT, podding time score; MA, maturity score; EM, emergence score; EV, early vigor score; PH, plant height. ^∗^*p* < 0.05, ^∗∗^*p* < 0.01, ^∗∗∗^*p* < 0.001.*

### SNP, Linkage Disequilibrium, and Population Structure

**FIGURE 1:**
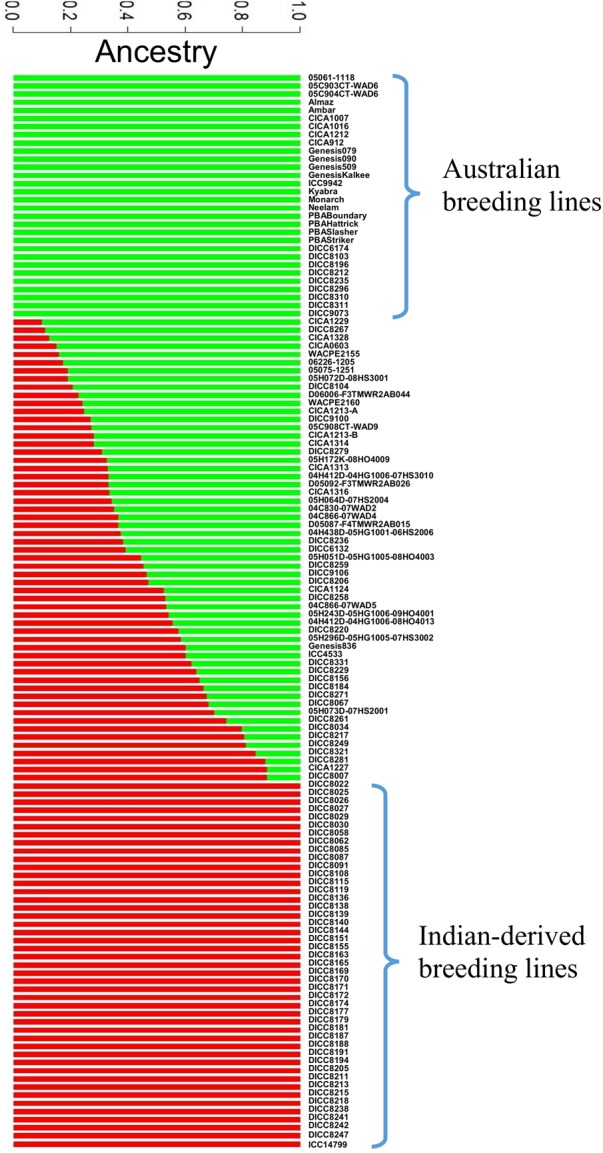
Population structure diagrams of the 132 genotypes. Results of population structure are shown when the numbers of estimated clusters is *k* = 2. The vertical bar is partitioned into red and green segments that represent the genotype’s estimated membership fractions.

A total of 144,777 homozygous SNPs were discovered in 132 genotypes (**Table 3**). The number of SNPs on each chromosome ranged from 25,323 on Ca4 to 4,740 on Ca8, partially reflecting the length of the chromosomes in the Kabuli 2.6.3 reference assembly. The extent of LD on each chromosome ranged from 4,000 kb on Ca3 to 150 kb on Ca6 with an average of 700 kb (**Table 3** and Supplementary Figure S2). The average extent of LD is almost seven times smaller than a previous study, in which mainly Australian-released chickpea varieties were used (Li et al., 2017). The short extent of LD in the 132 genotypes has the potential to enable higher mapping resolution. To avoid false positive results in association analysis, the population structure was investigated using 144,777 SNPs (**Figure 1**). The most likely number of groups (K) in the 132 genotypes was estimated to be two using a cross-validation method from the ADMIXTURE software. The red group in **Figure 1** is mainly the DICC lines (selected from ICRISAT breeding lines) consisting of progenies from the crosses of ICCV98503 × Moti, ICCV96836 × PBG5, ICCV96836 × ICC12004, and ICCV96836 × ICC3996. The green group in **Figure 1** consists of Australian-released varieties and advanced lines. Genotypes with a mixture of red and green have mixed ancestry from ICRISAT, ICARDA, and Australia.

**Table 3:** Summary of LD and SNPs used to estimate LD.

Chromosome	No. of SNPs	Density of SNPs (No. of SNPs/10 kb)	No. of SNPs used to estimate LD^1^	Mean *r*^2^	LD extent (kb)
Ca1	20,837	4.25	2,979	0.06	400
Ca2	11,181	3.01	2,826	0.03	200
Ca3	19,487	2.92	1,674	0.15	4,000
Ca4	25,323	4.30	7,106	0.02	200
Ca5	18,313	2.64	1,428	0.07	200
Ca6	15,620	2.37	2,469	0.05	150
Ca7	13,272	2.36	2,070	0.03	200
Ca8	4,740	2.38	933	0.06	200
Unassembled contigs	16,004	3.25	NA	NA	NA
Total/average	144,777		21,485	0.06	700

*^*1*^SNPs with high confidence (minimum five reads per genotype).*

### Genome-Wide Association Mapping

**FIGURE 2:**
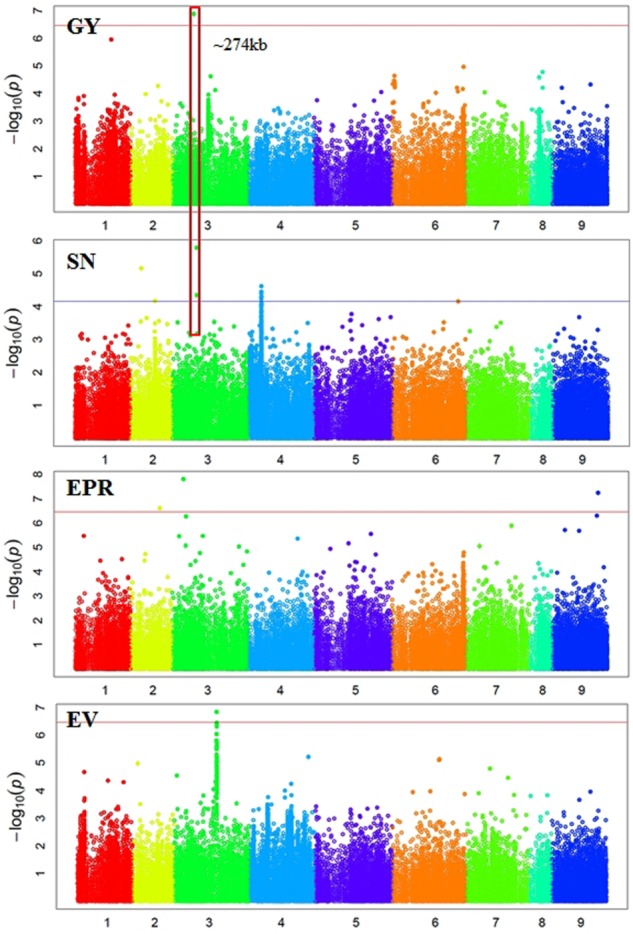
Manhattan plots showing GWAS results of grain yield (GY), seed number (SN), empty pod ratio (EPR), and early vigor score (EV). Each dot represents a SNP. The *x*-axis is the physical position of the SNP. Chromosomes are numbered from 1 to 8 while 9 represents all unassembled contigs. The red line is a significant threshold of *p*-value = 3.47e-07, equal to a level of 0.05 after Bonferroni correction. The blue line is a suggestive threshold of *p*-value = 1.0e-04. Regions containing SNPs significantly associated with different traits are highlighted with red rectangles.

**FIGURE 3:**
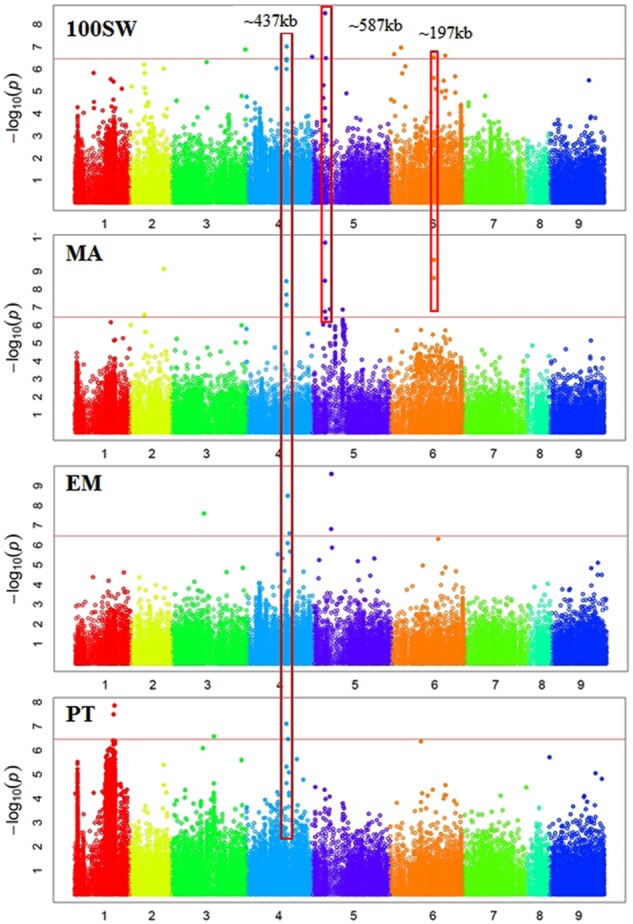
Manhattan plots showing GWAS results of hundred seed weight (100SW), maturity score (MA), emergence score (EM), and podding time score (PT). Each dot represents an SNP. The *x*-axis is the physical position of the SNP. Chromosomes are numbered from 1 to 8 while 9 represents all unassembled contigs. The red line is a significant threshold of *p*-value = 3.47e-07, equal to a level of 0.05 after Bonferroni correction. Regions containing SNPs significantly associated with different traits are highlighted with red rectangles.

In total, 38 SNPs were significantly (*p* < 3.45e-07) associated with six traits: GY, 100SW, EPR, PT, EM, and EV (Supplementary Table S1 and **Figures 2, 3**). One SNP, located in Ca3: 18,924,965, was significantly associated with GY (**Figure 2**). The closest gene near this SNP encodes a protein belonging to the ABC transporter B family/p-glycoprotein (PGP). Nine SNPs, located on Ca3, Ca4, Ca5, and Ca6, were significantly associated with 100SW (Supplementary Table S1 and **Figure 2**). Candidate genes located near these SNPs include two sugar transporters, two nodulin MtN21/EamA-like transporters, one Lateral Organ Boundaries (LOB) domain protein and several uncharacterized genes.

There were five SNPs significantly associated with PT, including two SNPs on Ca1, one SNP on Ca3, and two SNPs on Ca4 (Supplementary Table S1 and **Figure 3**). One was located on Ca4: 35,589,599 near a gene encoding a major latex protein (MLP), which promotes vegetative growth and delays flowering in Arabidopsis (Guo et al., 2011). Another significant SNP was located on Ca3: 38,173,722 close to a transcriptional factor squamosa promoter-binding-like protein 9 (SPL9). It was shown that SPL9 and SPL15 act redundantly in promoting the juvenile-to-adult phase transition in Arabidopsis (Schwarz et al., 2008).

There were 12 SNPs significantly associated with MA, including one SNP on Ca2, three SNPs on Ca4, six SNPs on Ca5, and two SNPs on Ca6 (Supplementary Table S1 and **Figure 3**). One of the significant SNPs was located on Ca5: 11,580,061, near the nodulin MtN21/EamA-like transporter which has been shown to be involved in auxin homoeostasis (Ranocha et al., 2013). Another significant SNP was located on Ca5: 12,166,907, near a sugar transporter gene with an important role in plant growth (Wobus and Weber, 1999). Six SNPs were significantly associated with EM, including one SNP on Ca3, three SNPs on Ca4, and two SNP on Ca5 (Supplementary Table S1 and **Figure 3**). One of the significant SNPs (Ca4: 35,589,599) was located near a gene encoding MLP, which promotes vegetative growth in Arabidopsis as described above (Guo et al., 2011). Two SNPs were significantly associated with EV (Supplementary Table S1 and **Figure 2**). One of the significant SNPs (Ca3: 38,177,160) was located near a gene encoding the transcriptional factor SPL9. It has been shown that SPL9 regulates leaf initiation negatively in Arabidopsis, leading to a shorter leaf plastochron, which is the time interval between two successive events of plant growth (Schwarz et al., 2008). There were no SNPs significantly (*p* < 3.45e-07) associated with SN, HI, DW, FT, or PH. This could be attributed to the lack of statistical power due to the small sample size (62–93 genotypes) employed in this study.

Some regions contain SNPs significantly associated with several different traits, which is an indication of a pleiotropic effect (**Figures 2, 3**). For example, a genomic region of ∼274 kb on Ca3 (18,924,965 to 21,660,191) contains a SNP significantly associated with GY and two SNPs weakly (*p*-value = 1.59e-06 and 4.49e-05) associated with SN. A ∼437 kb genomic region on Ca4 (35,589,599 to 36,026,910) contains eight SNPs, significantly associated with four traits: EM, maturity, PT, and 100SW. Four auxin-related genes, encoding one auxin efflux carrier protein (PIN3) and three nodulin MtN21/EamA-like transporters, are located in this region on Ca4. Another ∼587 kb genomic region on Ca5 (11,580,061 to 12,166,907) contains two SNPs, which were significantly associated with 100SW and MA. Five auxin-related genes, including one auxin influx transporter (LAX3) and four nodulin MtN21/EamA-like transporters, are located in this region. Two SNPs, significantly associated with both 100SW and maturity, were located in a 197 kb region on Ca6 (39,200,356 to 39,397,897) which contains two sugar transporters.

### Genomic Prediction

**FIGURE 4:**
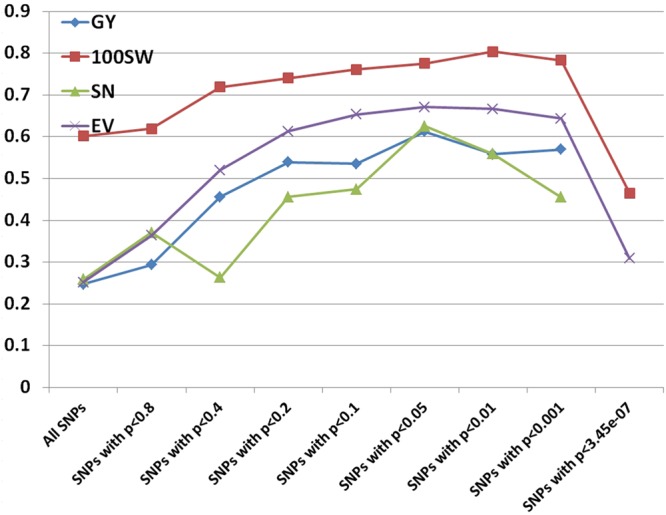
Prediction accuracies for grain yield (GY), hundred seed weight (100SW), seed number (SN), and early vigor score (EV) using different subsets of SNPs based on *p*-values from GWAS results.

Prediction accuracies for GY, 100SW, SN, and EV were estimated using RR-BLUP and different subsets of the SNPs based on *p*-values from GWAS results. Prediction accuracies increased when subsets of the SNPs based on a more stringent level of *p*-value were used (**Figure 4**). The increments plateaued in all four traits using subsets of SNPs with *p*-values between 0.05 and 0.01 and dropped dramatically at *p*-values of 3.45e-07 (equal to 0.05 after Bonferroni correction). The lowest prediction accuracies in three traits occurred when using all SNPs, which was probably due to noise introduced by non-causal variants as RR-BLUP shrikes each marker effect equally (de Los Campos et al., 2013). We also used BL and BRR to estimate prediction accuracies using subsets of SNPs. The results were similar to the RR-BLUP model.

## Discussion

Previous effort on breeding drought-tolerant chickpea has concentrated on accelerating flowering to escape terminal drought (Upadhyaya et al., 2012). This study showed that some India-derived genotypes with early podding trait aborted early onset pods in the Australian environments due to low temperatures in early spring. This suggests that it may be more relevant to focus on breeding for drought tolerance *per se* under Australia environments, traits such as water-use efficiency (Zaman-Allah et al., 2011; Kashiwagi et al., 2013), beneficial root traits (Zaman-Allah et al., 2011), stomatal conductance (Rehman et al., 2011), and osmotic adjustment (Morgan et al., 1991). As pointed out by Berger et al. (2016), the selection pressure for drought escape and drought tolerance *per se* is very different. Excessive use of the drought escape mechanism can compromise yield potential due to shorter life cycles to accumulate water and light resources. Thus a new breeding strategy is warranted, such as the integrated framework proposed by Berger et al. (2016).

### Auxin-Related Genes and Sugar Transporters Play an Important Role in Yield-Related Traits under Drought-Prone Environments

Several auxin-related genes, including PIN3, ABC transporter B family/PGP, and nodulin MtN21/EamA-like transporters, were found to be near SNPs significantly associated with GY, 100SW, PT, EM, and MA. Auxin (primarily indole-3-acetic acid) is a well-known phytohormone that plays a pivotal role in plant growth, seed development, and abiotic stress response (Zhao, 2010; Kazan, 2013; Locascio et al., 2014). A recent review paper summarized how plants coordinate auxin biosynthesis, transport, perception under osmotic stresses induced be drought, salinity (Naser and Shani, 2016). Auxin was found to enhance drought tolerance via the regulation of root architecture, expression of abiotic stress genes (*DREB2A* and *DREB2B*), ROS metabolism, and metabolic homeostasis in Arabidopsis (Shi et al., 2014). PIN3, belonging to the auxin efflux carrier protein family, have been characterized as important regulators involved in plant growth, phototropic response, and drought stress response (Ding et al., 2011; Zhang et al., 2012, 2013). A study in rice showed that drought, cold and heat stress affected the expression of genes involved in auxin signaling and polar transport, such as the PIN protein family (Du et al., 2013). Several studies have shown that PGP is involved in auxin transport through the plasma membrane and can stabilize the PIN protein family (Geisler and Murphy, 2006; Blakeslee et al., 2007; Titapiwatanakun et al., 2009; Zazimalova et al., 2010). Arabidopsis WAT1, a homolog of the nodulin MtN21/EamA-like transporter, was recently identified as a vacuolar auxin transporter required for auxin homoeostasis, a process that maintains an endogenous steady-state concentration of primary auxin (Ranocha et al., 2013). Several sugar transporters were found to be near SNPs significantly associated with 100SW and MA. Comprising hexose and sucrose transport proteins, the sugar transporters are members of the major facilitator superfamily. Sugar transporters play a key role in plant growth, source–sink partitioning, molecular signaling, and seed development, and are therefore important for optimal plant development and crop yield (Wobus and Weber, 1999; Wingenter et al., 2010; Doidy et al., 2012).

Using a bi-parental QTL mapping population, a “QTL-hotspot” region on Ca4 13,239,546 to 13,547,009 (based on the kabuli reference genome v1.0) was associated with at least seven traits including root traits, 100SW, PH, and days to flowering (Varshney et al., 2014b; Jaganathan et al., 2015; Kale et al., 2015). In this study, we did not identify SNPs from the “QTL-hotspot” region significantly associated with any traits. We identified a ∼437 kb genomic region on Ca4: 35,589,599 to 36,026,910 (Ca4: 37,933,355 to 38,412,853 based on the kabuli reference genome v1.0) containing eight SNPs significantly associated with four traits: EM, maturity, PT, and 100SW. Different 100SW QTL were identified between the two studies, which may be attributed to the different mapping populations examined.

### Confounding Effects of Self-Incompatibility with Pod Abortion under Drought

Three SNPs, located on Ca2, Ca3, and an unassembled contig, were significantly associated with EPR (Supplementary Table S1 and **Figure 2**). The kinesin-4 and self-incompatibility (SI) proteins were adjacent to two of the three significant SNPs. The kinesin-4 family plays an important role in cell elongation and has been shown to affect the length of siliques and seeds produced per silique in Arabidopsis (Kong et al., 2015). The SI protein (IPR010264) is highly homologous (with a total score of 141 and E value of 2e-32 using NCBI blastn) to a Medicago gene Medtr1g057250.1, which was well characterized in *Papaver rhoeas* (Foote et al., 1994; Wilkins et al., 2014). SI is a mechanism used by many flowering plants to prevent self-fertilization and inbreeding depression. Pollen from SI plants, carrying the same haplotype as the pistil, was rejected via the program cell death mechanism (Wilkins et al., 2014). Chickpea is generally a self-pollinating crop with an outcrossing rate of less than 2% (Toker et al., 2006); however, SI plants with empty pods yet viable pollen were observed in an F2 segregating population from a cross (H 82-5 × E100 ym) × Bhim with ∼22% of F2 SI plants (Lather and Dahiya, 1992). Pod abortion has long been thought to be introduced mainly by abiotic stress (Fang et al., 2010; Pang et al., 2017). Our findings, however, indicated that SI might have confounding effects with pod abortion under drought.

### Incorporating the Results of GWAS Increased Prediction Accuracy

Prediction models, employing variable selection procedures such as the LASSO, are considered to be better than RR-BLUP theoretically because they remove non-causal variants and variants not in LD with causal variants (Daetwyler et al., 2010; Meuwissen and Goddard, 2010). However, Wimmer et al. (2013) showed that LASSO failed to achieve its superiority and thus suggested to “*preselect markers according to biological prior information*.” Our study supports this assumption by showing that prediction accuracies were significantly improved using a subset of SNPs significantly (between *p* < 0.05 and *p* < 0.01) associated with traits. Several computer simulation studies (Meuwissen and Goddard, 2010; Ober et al., 2012) speculated that using large amounts of markers from WGRS may increase prediction accuracy, particularly in cases where the training population is distantly related to the prediction population. We argue that having more markers alone may not help to increase prediction accuracy, but it may help to identify the causal variants. If the prediction is done subsequent to the identification of causal variants, then the prediction may increase, as demonstrated in the current study, and thus the advantage of employing WGRS in GS can be realized.

Many studies have been published on the effect of marker density on prediction accuracy (Lorenzana and Bernardo, 2009; Vazquez et al., 2010; Weigel et al., 2010; Hoffstetter et al., 2016). Most have selected markers randomly or based on equal space and found that prediction accuracy increased when the number of markers increased, but reached a plateau depending on the extent of LD and the population size (Vazquez et al., 2010). A few studies selected markers based on biological prior information (Hoffstetter et al., 2016; Kooke et al., 2016; Spindel et al., 2016). Compared to using all markers in the RR-BLUP model, prediction accuracies doubled by using a subset of markers with significant association with grain yield in wheat (Hoffstetter et al., 2016). Another GS study in rice also showed that prediction accuracies were 7.0%–29.8% higher based on RR-BLUP with all markers and markers (selected from GWAS) fitted as fixed effects compared to that based on RR-BLUP with all markers alone (Spindel et al., 2016). Using different models, a recent GS study in chickpea indicated that prediction accuracies of yield under rainfed environments ranged from 0.148 to 0.186, which is similar (0.25) to this study using all 144,777 SNPs, but much smaller (0.56–0.61) than when using a subset of SNPs significantly associated with yield. We speculate that prediction accuracy may increase if an approach described here is adopted.

Training population size is an important factor in GS. Several studies have been conducted to investigate the optimum size for a training population in plants. Generally, the accuracy of estimated marker effects increases as the sample size increases (Albrecht et al., 2011; Endelman et al., 2014). Compared to other prediction models, one simulation study showed that RR-BLUP is robust with a small training population size even as low as *n* = 75, with diminishing benefits between *n* = 125 and *n* = 300 (Lorenz, 2013). Riedelsheimer and Melchinger (2013) reached a similar observation and recommended to allocate more resources to the selection candidates (prediction set) instead of the training population when budget is fix. Compared to a GS study in chickpea conducted by Roorkiwal et al. (2016), the training population size in this study is relatively small. Because the main objective of this study is to test the prediction accuracy based on subsets of significant SNPs. The result of this study should hold since the size of the training population was the same in different subsets of SNPs. For real breeding application, such as selecting candidate genotypes without phenotypic data, larger training populations should be used to increase prediction accuracy. Additionally, the training population needs to be updated regularly to maintain a close relationship with selection candidates (Neyhart et al., 2017).

Grain yield is a complex trait controlled by numerous genes with small effect. We found only one SNP significantly associated with grain yield in this study, probably due to limited statistical power to identify genes which underline complex traits. Even if all yield-related genes could be identified using a larger sample size, pyramiding favorable alleles from all genes into a single genotype using traditional marker-assisted selection or transgenic approaches would be extremely difficult. The superiority of the GS approach is that it can use all marker information simultaneously and thus circumvent the complex process of pyramiding. That is not to say that GWAS and marker-assisted selection do not have a place in molecular breeding; for example these approaches are useful for targeting simple traits (Mendelian traits) such as disease resistance (Varshney et al., 2014a; Li et al., 2017). This study also shows that incorporating the results of GWAS into the prediction model can significantly increase prediction accuracy. However, this gain of prediction accuracy is only examined in a cross-validation scheme. Further study is needed to investigate whether this result holds true when this approach is applied to selection candidates.

## Author Contributions

YL and TS conceived the study; YL performed the GWAS and GS analysis; JB contributed to sequencing; PR analyzed the sequencing data; DE supervised sequencing-data analysis; KS, JP, TK, and TC contributed to phenotyping; YL wrote the manuscript and all the authors read and approved the manuscript.

## Conflict of Interest Statement

The authors declare that the research was conducted in the absence of any commercial or financial relationships that could be construed as a potential conflict of interest.

## Footnotes

1 http://www.cicer.info/databases.php
