# Supplementary material for: Investigating Drought Tolerance in Chickpea Using Genome-Wide Association Mapping and Genomic Selection Based on Whole-Genome Resequencing Data
Source: Front Plant Sci. 2018 Feb 19;9:190. doi: 10.3389/fpls.2018.00190 (PMC5825913; doi:10.3389/fpls.2018.00190)
Supplement: Supplementary file 1 [file Table_1.DOCX]

Supplementary Material

**Investigating Drought Tolerance in Chickpea Using Genome-wide Association Mapping and Genomic Selection Based on Whole-genome Resequencing Data**

Yongle Li^1*^, Pradeep Ruperao^2^, Jacqueline Batley^3^, David Edwards^3^, Tanveer Khan^3^, Timothy D. Colmer^3^, Jiayin Pang^3^, Kadambot H.M Siddique^3^, Tim Sutton^5, 1^

*** Correspondence:** yongle.li@adelaide.edu.au

**Table S1** Summary of significant SNP-trait associations

| Trait | SNP ID | Location^1^ | P-value | Closest flanking genes |
| --- | --- | --- | --- | --- |
| GY | Ca70402 | Ca3:18,924,965 | 1.31E-07 | ABC transporter B family/ p-glycoprotein (PGP) protein, uncharacterized protein |
| 100SW | Ca92792 | Ca3:66,074,996 | 1.34E-07 | uncharacterized proteins |
|  | Ca140120 | Ca4:36,026,910 | 9.95E-08 | tetratricopeptide repeat protein, uncharacterized protein |
|  | Ca167095 | Ca5:96,502 | 2.90E-07 | pentatricopeptide repeat-containing protein, LOB domain-containing protein |
|  | Ca174511 | Ca5:11,580,061 | 3.11E-09 | two nodulin MtN21 /EamA-like transporters |
|  | Ca214172 | Ca6:9,976,746 | 1.10E-07 | S-acyltransferase, gamma-aminobutyrate transaminase |
|  | Ca174812 | Ca5:12,166,907 | 3.24E-07 | two sugar transporters |
|  | Ca206729 | Ca6:3,732,289 | 2.19E-07 | pentatricopeptide repeat-containing protein, uncharacterized protein |
|  | Ca247725 | Ca6:39,271,136 | 3.04E-07 | two uncharacterized proteins |
|  | Ca263202 | Ca6:49,490,394 | 2.56E-07 | shikimate O-hydroxycinnamoyltransferase, uncharacterized protein |
| EPR | Ca52884 | Ca2:26,054,696 | 2.48E-07 | uncharacterized protein |
|  | Ca64147 | Ca3:9,796,206 | 1.55E-08 | Kinesin-4 subfamily |
|  | Ca344248 | Ca9:40,908,266 | 5.75E-08 | Plant self-incompatibility protein, uncharacterized protein |
| PT | Ca26012 | Ca1:34,856,055 | 3.14E-08 | uncharacterized serine-rich protein, uncharacterized protein |
|  | Ca27769 | Ca1:35,710,766 | 1.34E-08 | lipid-transfer protein 8-like, uncharacterized protein |
|  | Ca80083 | Ca3:38,173,722 | 2.66E-07 | squamosa promoter-binding-like protein 9, heterogeneous nuclear ribonucleoprotein A1 |
|  | Ca139795 | Ca4:35,852,249 | 7.73E-08 | long chain acyl-CoA synthetase 9, uncharacterized protein |
|  | Ca139425 | Ca4:35,589,599 | 3.44E-07 | Auxin efflux carrier family (PIN3), MLP-like protein |
| MA | Ca55941 | Ca2:30,550,216 | 2.43E-11 | UDP-glucosyltransferase protein, alpha-1,4 glucan phosphorylase L isozyme |
|  | Ca139795 | Ca4:35,852,249 | 8.22E-10 | long chain acyl-CoA synthetase 9, uncharacterized protein |
|  | Ca139935 | Ca4:35,930,557 | 4.46E-10 | aldehyde dehydrogenase family 3, BTB/POZ domain-containing protein At1g21780 |
|  | Ca140120 | Ca4:36,026,910 | 3.91E-09 | tetratricopeptide repeat protein, uncharacterized protein |
|  | Ca172479 | Ca5:9,970,803 | 5.55E-08 | trans-cinnamate 4-monooxygenase, uncharacterized protein |
|  | Ca174075 | Ca5:11,349,381 | 3.75E-11 | sodium-coupled neutral amino acid transporter 6, uncharacterized protein |
|  | Ca174188 | Ca5:11,408,248 | 3.08E-08 | Peptide transporter PTR3-A, uncharacterized protein |
|  | Ca174511 | Ca5:11,580,061 | 5.15E-13 | two nodulin MtN21 /EamA-like transporters |
|  | Ca174812 | Ca5:12,166,907 | 1.36E-08 | two sugar transporters |
|  | Ca177217 | Ca5:15,683,520 | 7.42E-08 | receptor-like protein kinase HSL1, epidermal patterning factor-like protein |
|  | Ca247724 | Ca6:39,271,118 | 1.48E-11 | two uncharacterized proteins |
|  | Ca247725 | Ca6:39,271,136 | 1.27E-10 | two uncharacterized proteins |
| EM | Ca76901 | Ca3:28,288,711 | 6.11E-09 | serine/threonine-protein phosphatase, uncharacterized protein |
|  | Ca139425 | Ca4:35,589,599 | 5.10E-09 | Auxin efflux carrier family (PIN3), MLP-like protein |
|  | Ca139795 | Ca4:35,852,249 | 4.37E-09 | long chain acyl-CoA synthetase 9, uncharacterized protein |
|  | Ca143108 | Ca4:37,433,797 | 5.10E-09 | GATA transcription factor 12 (light-responsive transcription), 31 kDa ribonucleoprotein |
|  | Ca177217 | Ca5:15,683,520 | 1.79E-09 | receptor-like protein kinase HSL1, epidermal patterning factor-like protein |
|  | Ca177291 | Ca5:15,967,108 | 2.29E-09 | DNA topoisomerase 1, uncharacterized protein |
| EV | Ca79976 | Ca3:38,044,373 | 1.45E-07 | transcription factor bHLH130, uncharacterized protein |
|  | Ca80089 | Ca3:38,177,160 | 3.45E-07 | squamosa promoter-binding-like protein 9, heterogeneous nuclear ribonucleoprotein A1 |

GY=grain yield, 100SW=hundred seed weight, EPR=empty pod ratio, PT=podding time score, MA=maturity score, EM=emergence score, EV=early vigour score

^1^ Location is based on the kabuli reference genome version 2.6.3 (http://cicer.info/databases.php)

**Figure S1** The distributions of the BLUP values of the 12 yield and yield-related traits. GY=grain yield, %100SW=hundred seed weight, SN=seed number, EPR=empty pod ratio, HI=harvest index, DW=Biomass dry weight, FT=flowering time score, PT=podding time score, MA=maturity score, EM=emergence score, EV=early vigour score, PH=plant height


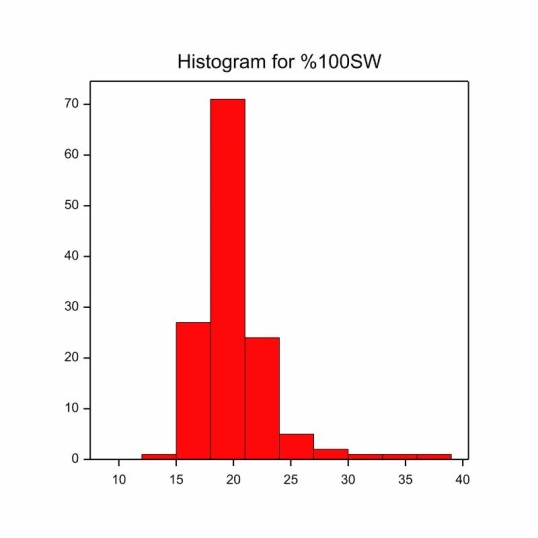

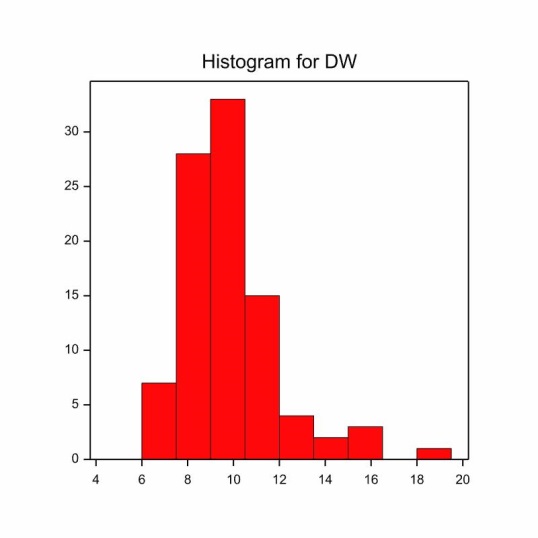

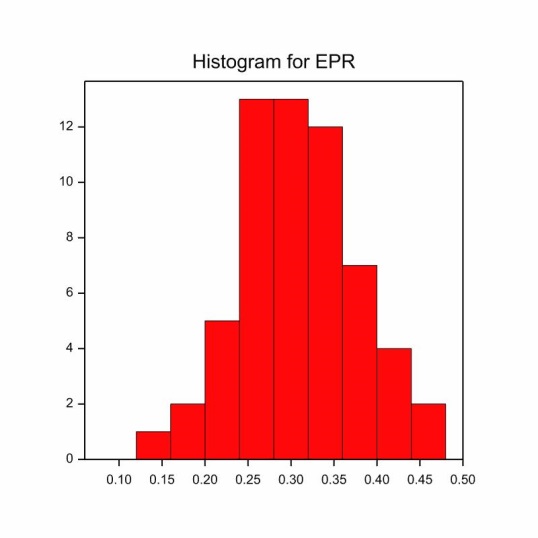

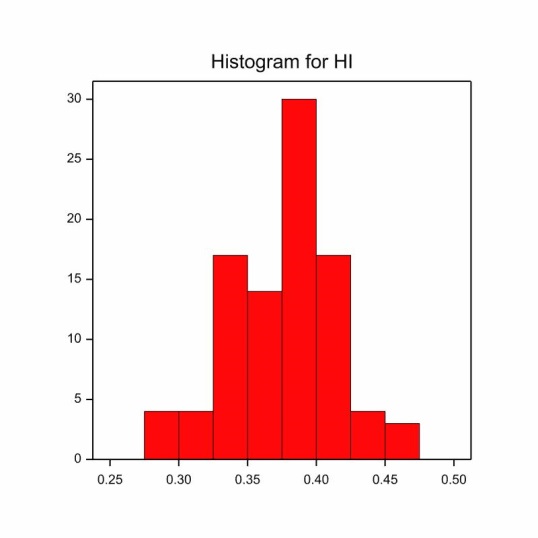

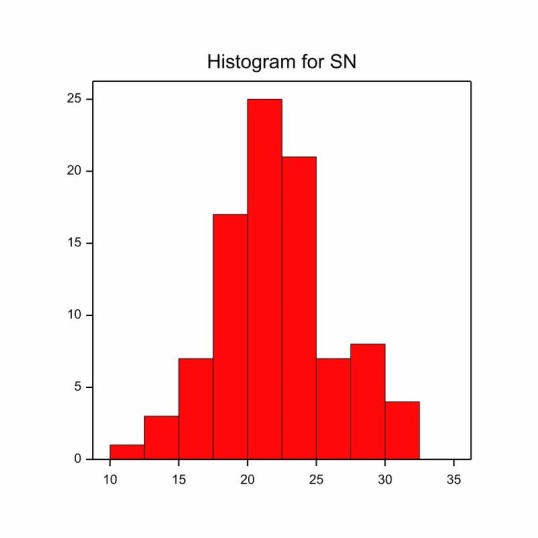

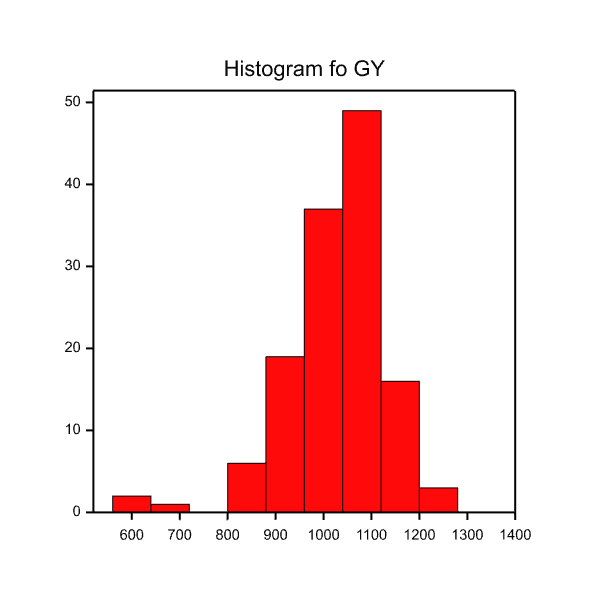

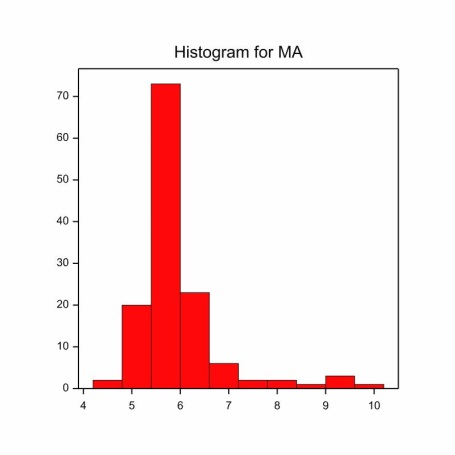

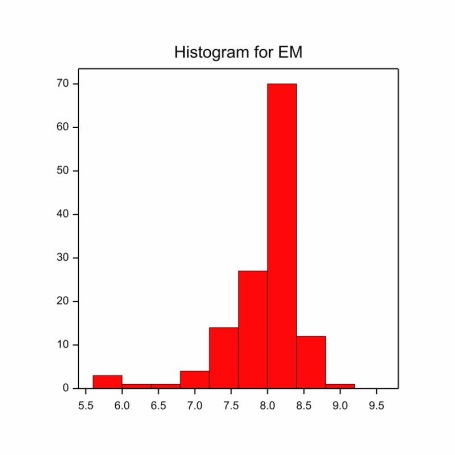

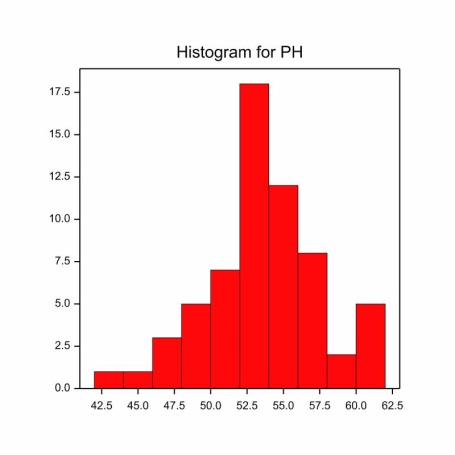

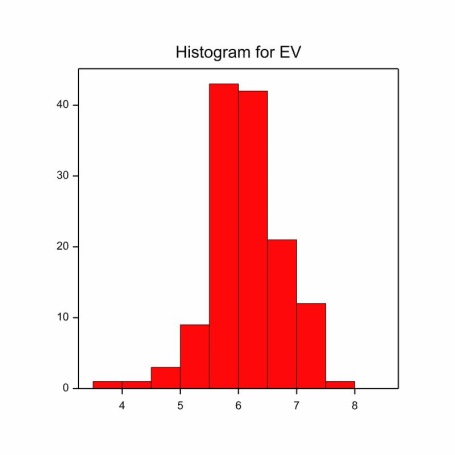

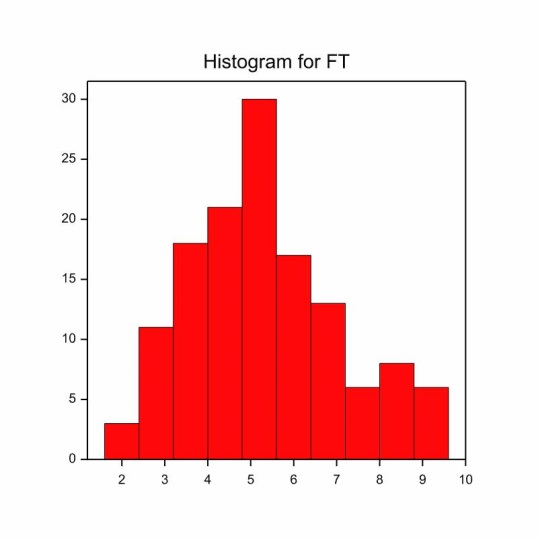

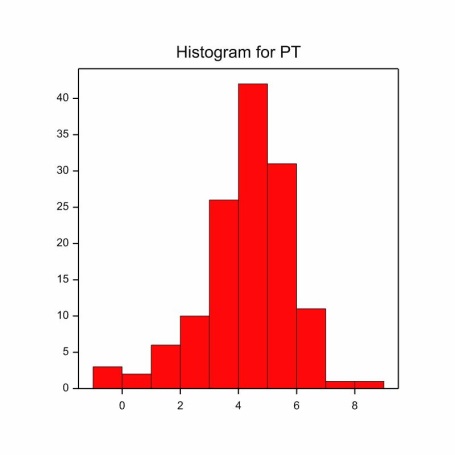


**Figure S2** LD extent in the eight chromosomes of the 132 chickpea genoytpes. Horizontal axis means the physical distance (bp), vertical axis means the LD between SNP markers measured as *r*2. The blue lines represent the LD extent threshold (*r*2 = 0.2). The red curves are the trend of LD extent fitted using the mutation-drift-equilibrium model.

**
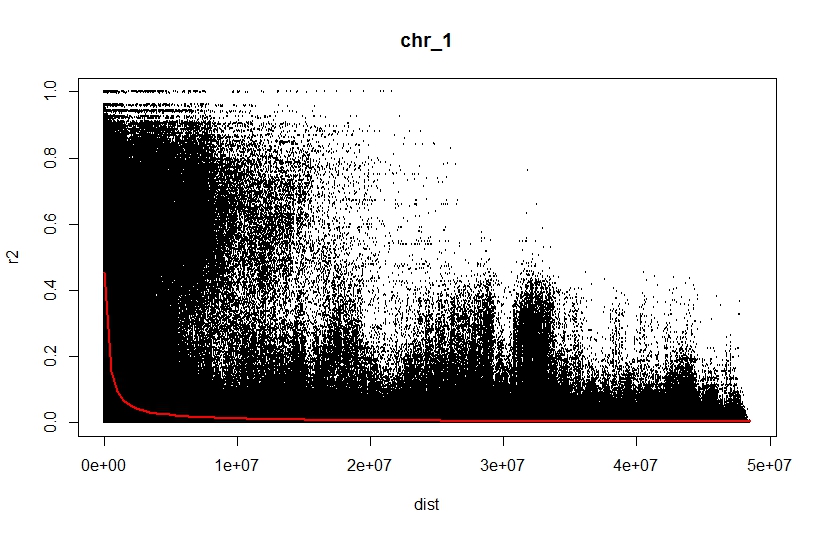
**

**
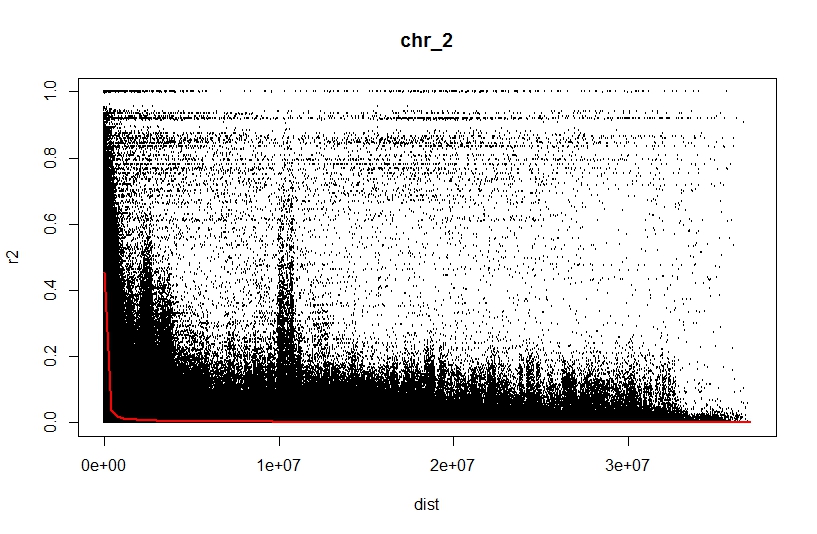
**

**
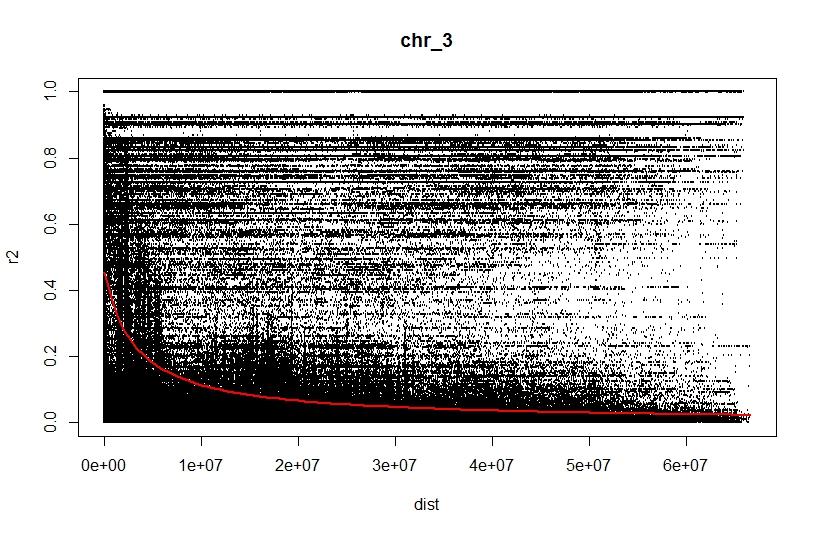
**

**
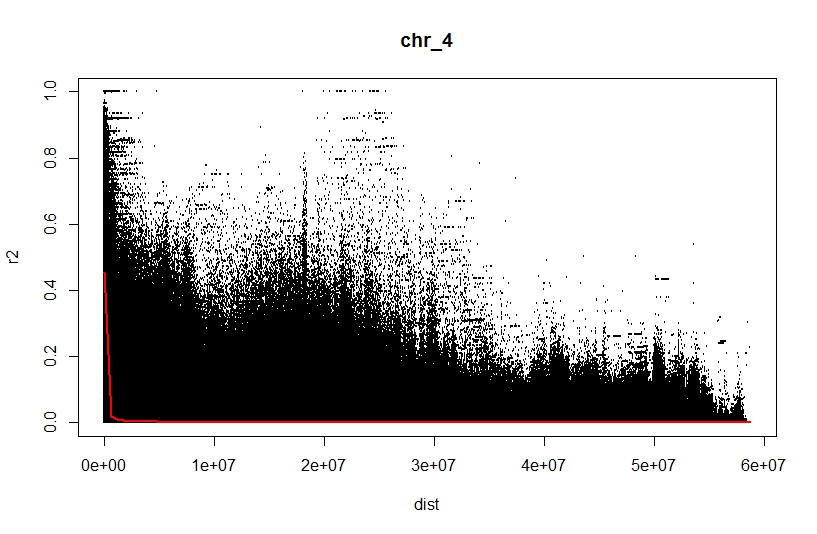
**

**
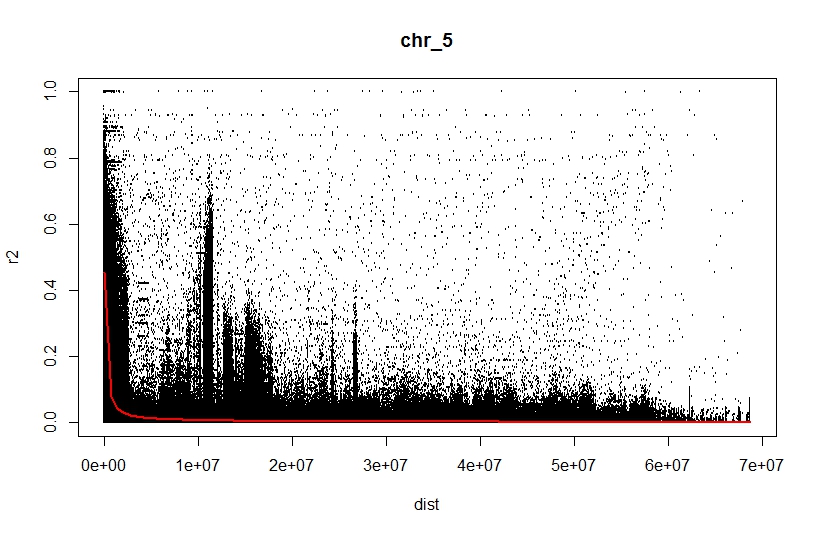
**

**
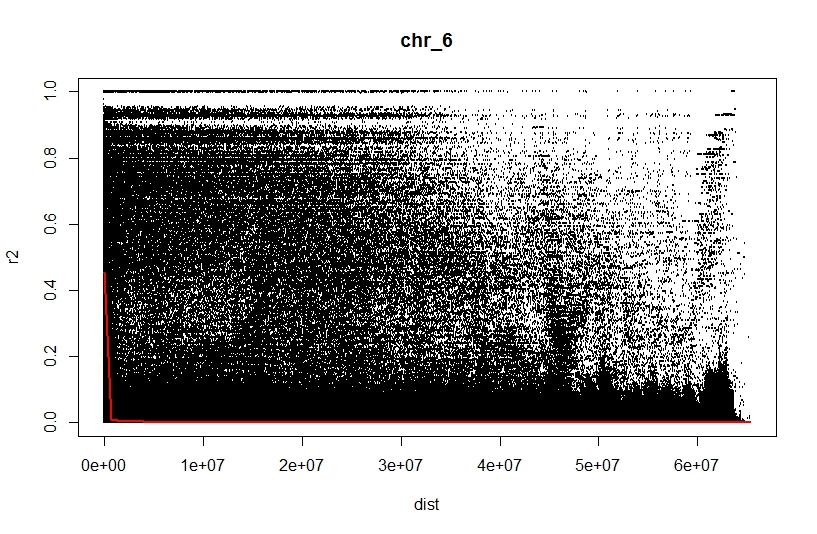
**

**
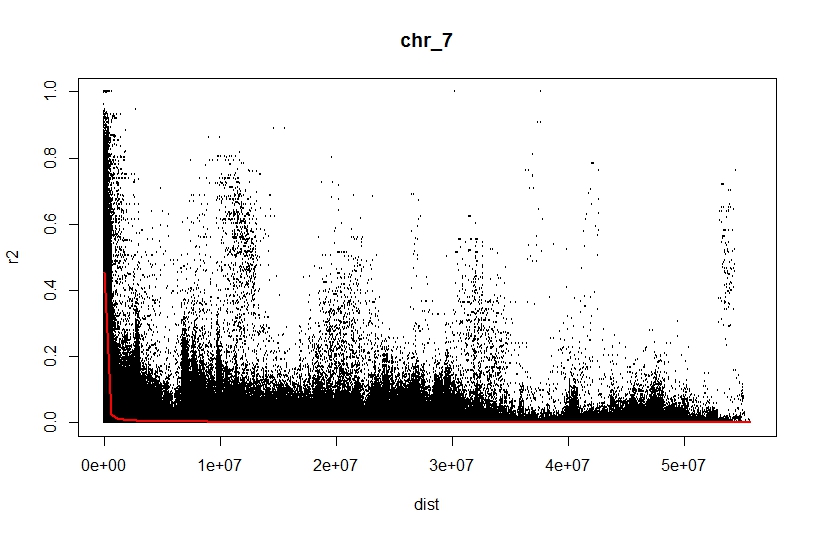
**

**
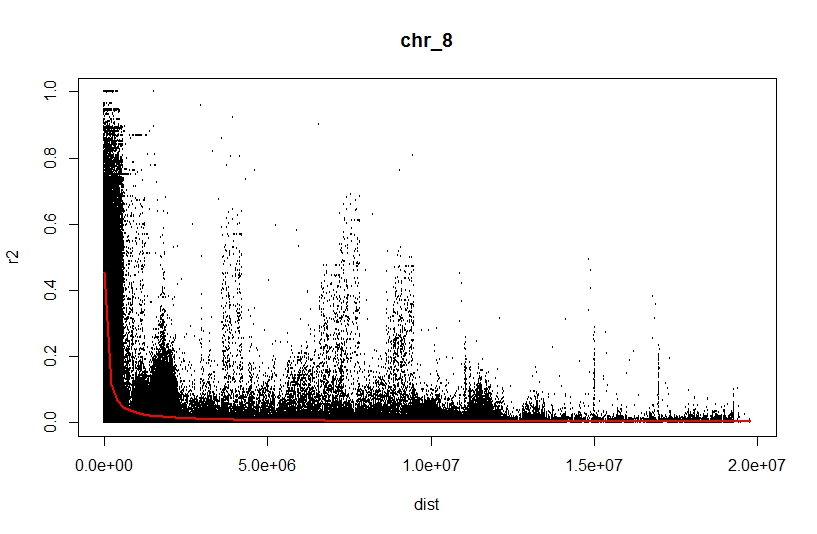
**
